# Metabolism and Chronic Inflammation: The Links Between Chronic Heart Failure and Comorbidities

**DOI:** 10.3389/fcvm.2021.650278

**Published:** 2021-05-05

**Authors:** Zhiwei Li, Hongmei Zhao, Jing Wang

**Affiliations:** Department of Pathophysiology, State Key Laboratory of Medical Molecular Biology Institute of Basic Medicine, Chinese Academy of Medical Sciences, School of Basic Medicine, Peking Union Medical College, Beijing, China

**Keywords:** heart failure, comorbidities, metabolism, chronic inflammation, reactive oxygen species, mitochondria

## Abstract

Heart failure (HF) patients often suffer from multiple comorbidities, such as diabetes, atrial fibrillation, depression, chronic obstructive pulmonary disease, and chronic kidney disease. The coexistance of comorbidities usually leads to multi morbidity and poor prognosis. Treatments for HF patients with multi morbidity are still an unmet clinical need, and finding an effective therapy strategy is of great value. HF can lead to comorbidity, and in return, comorbidity may promote the progression of HF, creating a vicious cycle. This reciprocal correlation indicates there may be some common causes and biological mechanisms. Metabolism remodeling and chronic inflammation play a vital role in the pathophysiological processes of HF and comorbidities, indicating metabolism and inflammation may be the links between HF and comorbidities. In this review, we comprehensively discuss the major underlying mechanisms and therapeutic implications for comorbidities of HF. We first summarize the potential role of metabolism and inflammation in HF. Then, we give an overview of the linkage between common comorbidities and HF, from the perspective of epidemiological evidence to the underlying metabolism and inflammation mechanisms. Moreover, with the help of bioinformatics, we summarize the shared risk factors, signal pathways, and therapeutic targets between HF and comorbidities. Metabolic syndrome, aging, deleterious lifestyles (sedentary behavior, poor dietary patterns, smoking, etc.), and other risk factors common to HF and comorbidities are all associated with common mechanisms. Impaired mitochondrial biogenesis, autophagy, insulin resistance, and oxidative stress, are among the major mechanisms of both HF and comorbidities. Gene enrichment analysis showed the PI3K/AKT pathway may probably play a central role in multi morbidity. Additionally, drug targets common to HF and several common comorbidities were found by network analysis. Such analysis has already been instrumental in drug repurposing to treat HF and comorbidity. And the result suggests sodium-glucose transporter-2 (SGLT-2) inhibitors, IL-1β inhibitors, and metformin may be promising drugs for repurposing to treat multi morbidity. We propose that targeting the metabolic and inflammatory pathways that are common to HF and comorbidities may provide a promising therapeutic strategy.

## Introduction

Heart failure (HF) is a global public health problem that affects more than 26 million people worldwide and causes a heavy health burden (1, 2). The prevalence of HF was 1.3% in Chinese adults (an estimated 13.7 million), in which 23% of patients had HF with preserved ejection fraction (EF), (HFpEF), 23% had HF with middle-range EF (HFmrEF), and about 54% had reduced ejection fraction (HFrEF) (3). Due to the aggravation of aging, the incidence of HF is rising, and HF is associated with increased mortality, morbidity, and hospitalization (4).

**Figure 1:**
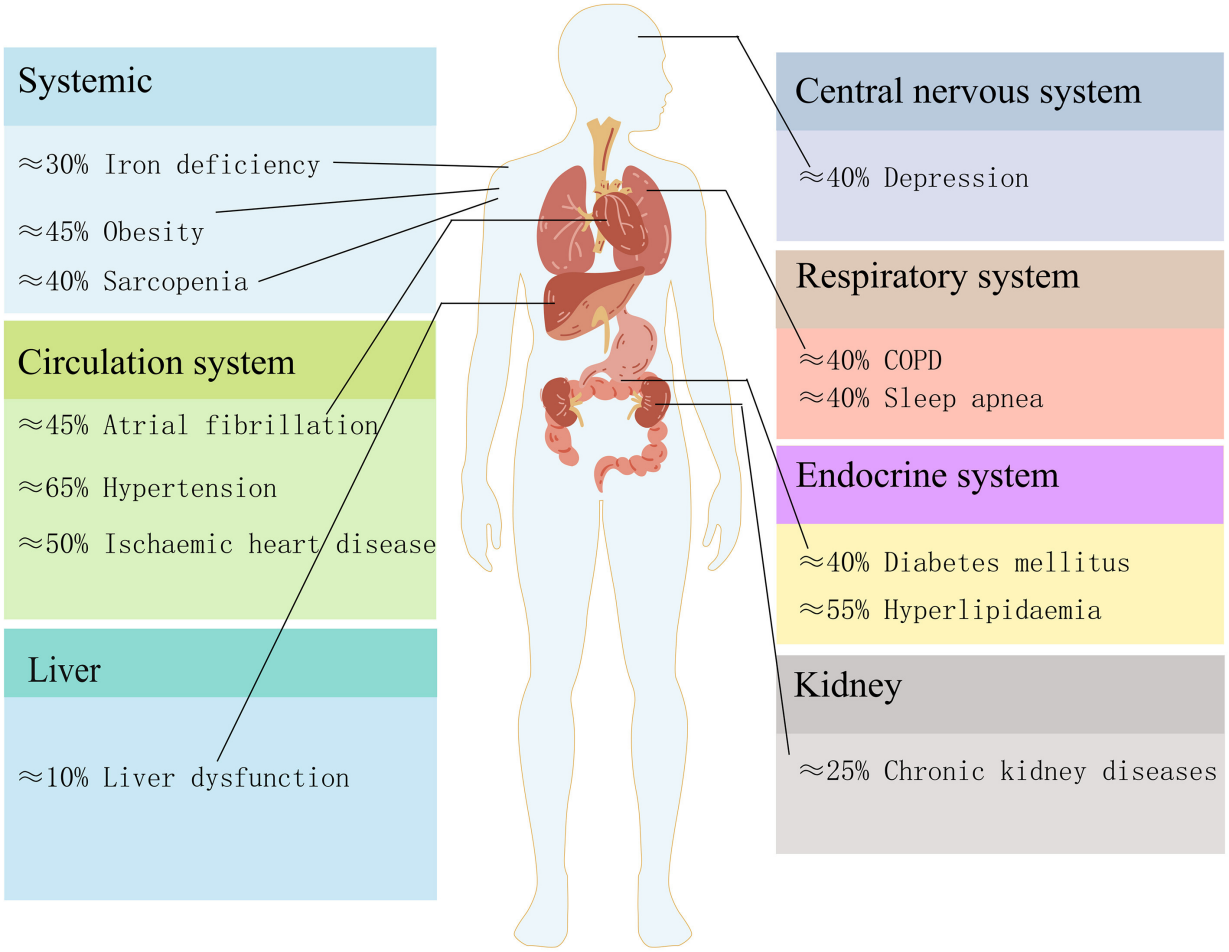
The estimated prevalence of heart failure comorbidities in different organs and systems.

HF often coexists with multiple comorbidities. The reported prevalence of comorbidities varied with HF severity (5). As shown in Figure 1, we summarized the prevalence of major comorbidities according to the different organs and systems involved, such as hypertension (65%), atrial fibrillation (45%), chronic obstructive pulmonary disease (COPD)/asthma(40%), iron deficiency (30%), diabetes (40%), chronic kidney diseases (CKD) (25%), obesity (45%) (6, 7), ischaemic heart disease (50%), hyperlipidaemia (55%) (8), depression (40%) (9–11), sleep apnea (40%) (12), sarcopenia (40%) (13) and liver dysfunction (10%) (14). The high prevalence of multi morbidity is associated with poor prognosis and heavy heath burdens, and therapy for multi morbidity in HF is still a challenge (15). However, the treatment of comorbidities may have cardiovascular side effects. Therefore, understanding the underlying mechanisms and finding potential strategies for both HF and comorbidities is worthwhile.

Metabolism and inflammation play an essential role in the pathophysiology of HF and its associated comorbidities, which may be the link between them. In this review, we summarized the role of metabolism and inflammation in HF and its most common comorbidities, and review their possible links, including shared risk factors, signal pathways, and therapeutic targets.

## Metabolic Remodeling From Normal to Failing Heart is Both Cause and Effect of Heart Failure

**Figure 2:**
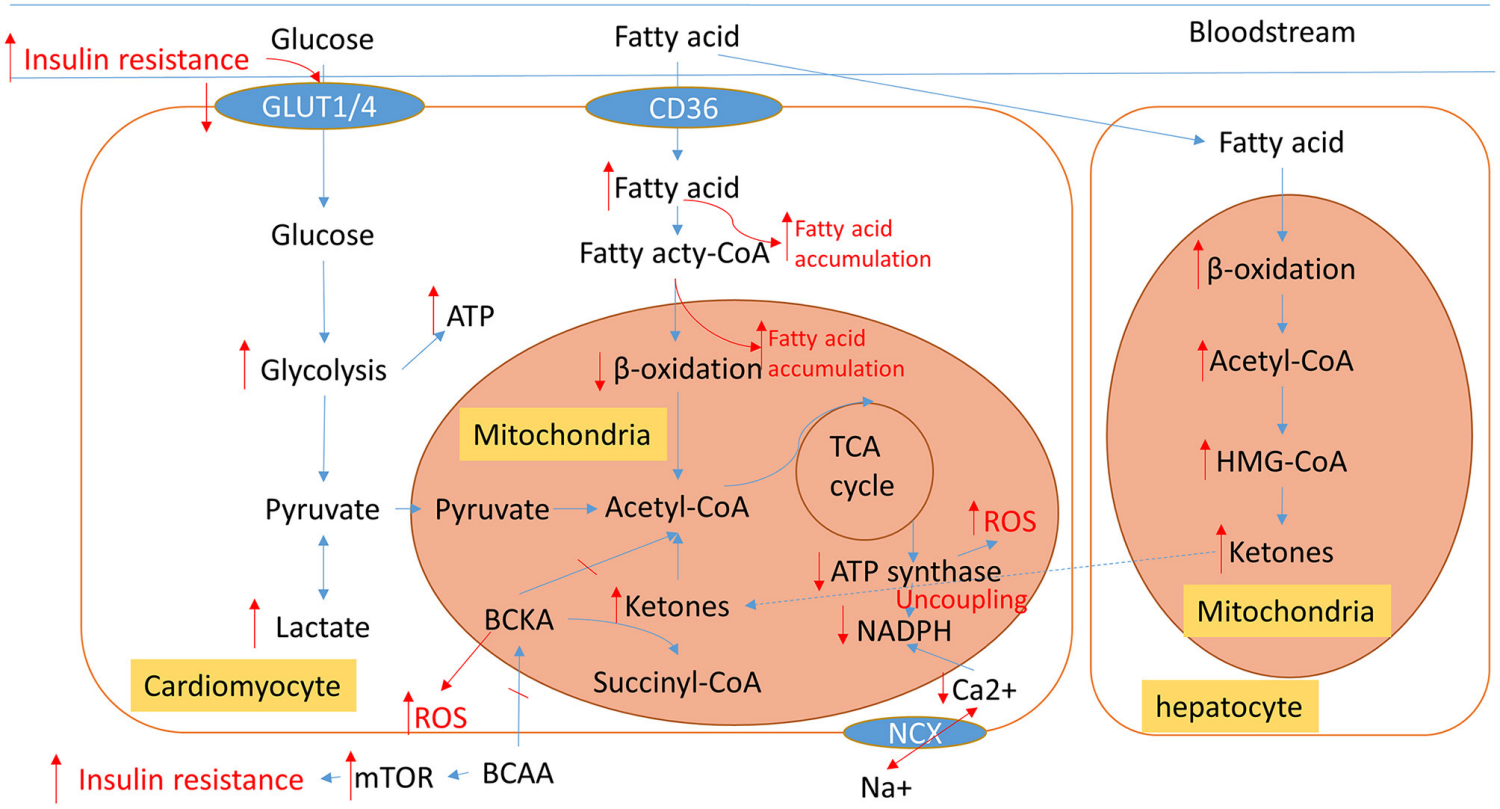
An overview of normal physiological metabolic processes and the pathological metabolic remodeling characteristic of HF. Blue arrows show normal cardiac metabolic processes. The altered metabolic processes of HF are displayed in red; Arrows indicate changed metabolic intermediates and products. TCA, tricarboxylic acid; BCCA, branched-chain amino acids; BCKA, branched-chain alpha-keto acids; ROS, reactive oxygen species; NCX, Na^+^/Ca^2+^ exchanger.

The heart requires a high rate of ATP production and turnover to fuel its continuous mechanical work, and it has become common knowledge that the failing heart is an “engine out of fuel” (16). We give an overview of the pathological cardiac metabolic remodeling from physiological condition to heart failure in Figure 2, including glucose, fatty acid (FA), amino acid, and ions metabolisms. These metabolic changes all affect cardiac energy metabolism either by directly participating in or indirectly regulating mitochondrial metabolism.

### Normal Cardiac Energy Metabolism Has Compensatory Capacity

Under normal physiological conditions, the heart cycles about 6 kg of ATP every day (16). Regulation of cardiac energy metabolism is through substrate alteration. The substrate mainly consists of fatty acids (FAs), glucose, pyruvate, lactate, and ketone bodies. Glucose and fatty acid metabolism are major contributors to cardiac energy metabolism.

At rest, about 15–25% of the heart's maximum energy loading capacity is used (17). The cardiac energy metabolic pathway can be altered in only a few seconds through substrate alterations when shifting from rest to acute stress such as exercise or ischemia, or after glycogen stores have been depleted when fasting. In the normal heart, about 60–90% (depending on energy demand) of the cardiac energy budget is produced by FA β-oxidation, and the rest is produced by the pyruvate and tricarboxylic acid (TCA) cycle (18). Under non-ischemic conditions, more than 95% of the ATP in the normal heart comes from oxidative phosphorylation of FAs, glucose, and lactate in mitochondria, while in a fasting state, as the energy demand increases, there is a substrate shift from FAs to glucose (FAs produce about 70% of the ATP and glucose produces 20%) (17).

There is an auto-balance mechanism of glucose and fatty acid oxidation (FAO) pathways in the energy substrate that called the “Randle cycle,” in which the activation of FAO would inhibit glucose uptake, whereas the increased utilization of glucose inhibits FAO, and inhibiting FAO increase glucose oxidation compensatorily (19). This regulation is mainly through an increase in plasma insulin level and the activation of the AMP-activated protein kinase (AMPK) pathway. Insulin would increase glucose uptake and activate the phosphatidylinositol 3-kinase (PI3K)/AKT pathway, and finally increases myocardial contractility. Glucose uptake is an insulin-dependent process because glucose transporters (GLUT1/GLUT4) are sensitive to insulin. The activation of AMPK promotes both FA and glucose oxidation which increases cardiac energy. Moreover, AMPK inhibits ATP-consuming processes like protein synthesis (20).

### Altered Energy Metabolic Substrate Utilization Is the Major Metabolic Remodeling in Heart Failure

The metabolic remodeling in the failing heart is similar to the alterations from the non-ischemic to the ischemic condition, as mentioned above, and may well be a protective compensatory mechanism to use more of its capacity. However, long term sustained high energy loading would cause some toxic substances to accumulate, which in turn may contribute to the progress of HF and comorbidities. In most cases, FAO decreases and glycolysis increases rapidly in HF, except for advanced and diabetic HF where FAO increases (18, 21, 22), this is because mitochondrial dysfunction in HF causes decreased expression and activity of enzymes associated with mitochondrial FAO (23). Several key enzymes of FAO are regulated by transcript factor peroxisome proliferator-activated receptors (PPARs). The decrease of FAO could be mainly explained by the activation of PPARγ and reduced activity of PPARα (18). Insulin plays an important role in substrate shift progression. Therefore, in cases of insulin resistance, such as diabetic HF or advanced HF, FAO is increased by activating PPARα signaling (22). Myocardial uptake of FA usually increases in HF. The imbalance of increased FA uptake and impaired utilization of FAs in HF results in FA accumulation. Accumulated FAs cause lipotoxicity and worsen HF by promoting mitochondrial dysfunction and apoptosis, and contributes to the development of insulin resistance (18).

Targeting the FAO pathway is an emerging treatment for HF (24), but the significance of the shift from FAO to glucose metabolism remains controversial and there have two opposite therapeutic strategies: inhibit or facilitate FA utilization. The two therapeutic strategies are not contradictory because they both reduce the cardiac accumulation of FAs, one is by reducing the uptake of FAs and the other is by increasing the catabolism of FAs. Drugs targeted inhibition of FAO may be classified into 3 categories: (1) β-oxidation inhibition, such as malonyl-CoA decarboxylase inhibitors, (2) mitochondrial FA uptake inhibition, such as the carnitine palmitoyl transferase 1 inhibitor (CPTI), (3) plasma membrane FA uptake reduction by inhibiting related proteins, such as in the case of CD36 (the major FA transporter) or fatty acid-binding protein (FABP). CD36 inhibitor is still under preclinical investigations. However, considering that glucose provides less capacity for energy production than FAs (one FA molecule produces 120–130 ATP, while one glucose molecule produces 30–32 ATP) (22), there is an opposite opinion, which asserts that the heart reverting back to using FA may have therapeutic value for HF, such as by targeting GLUT4 to inhibit glycolysis or activate the AMPK pathway by phosphorylation to increase FAO. Studies have confirmed that reverting to the use of FA has a cardio protective effect (22, 25). Restoration of FAO could improve heart function, possibly via reduced cardiac lipotoxicity (26).

Mitochondria are a physiological source of reactive oxygen species (ROS). They are generated in the electron transport chain (ETC) during respiration, and eliminated by NADPH dependent enzyme systems, forming a “redox-optimized ROS balance” (19). The deficit in energy would cause the uncoupling of oxidative phosphorylation, and cause an increase in reactive oxygen species (ROS) and oxidative stress (27). ROS, in return, inactivates several enzymes of the TCA cycle (19).

In addition, liver energy metabolism also participates in the process of HF. Ketone bodies synthesized in liver mitochondria, especially β-hydroxybutyrate, the so-called super fuel, are more efficient than FAs or glucose. The failing heart adaptively consumes more ketone bodies (28) and this is believed to be beneficial (23, 29).

### Amino Metabolism Dysfunction Indirectly Affects Cardiac Energy Metabolism

More glutamine is consumed in HF because it is the most abundant secreted amino acid (28), but branched-chain amino acids (BCAAs) played a more important role in HF. In healthy individuals, BCAAs are essential nutrition for mitochondrial biogenesis, and dietary supplementation of BCAAs has cardio protective effects (30–32). However, BCAA catabolic metabolism is impaired in HF, leading to the accumulation of BCAAs and branched-chain alpha-keto acids (BCKAs) (33). The accumulated BCAAs and their catabolic intermediates have a cardiotoxic effect. BCAA accumulation could result in insulin resistance by activating the mTOR pathway (34, 35), and accumulated BCKAs would increase reactive oxygen species (ROS) (36). Furthermore, BCAA is reported to be a potential therapeutic target for HF (37). BCAAs are not a major source of cardiac energy (below 5%) (28). but may have important indirect regulatory roles in energy metabolism as they affect mitochondrial biogenesis and BCAA toxicity affects energy metabolism.

### Ion Metabolism Induces Heart Failure by Regulating Energy Metabolism

Sodium (Na^+^) and calcium (Ca^2+^) ions are closely linked to HF. Elevated intracellular Na^+^ can lead to cardiac energy metabolic shift from FAO to glycolysis (38). The renin-angiotensin-aldosterone (RAAS) system has evolved to retain Na^+^ homeostasis and RAAS-blockers have been widely used in HF therapy. In HF, tubular cells are often hypertrophic and Na^+^ reabsorption increases. Sodium-glucose transporter-2 (SGLT-2) is a recently discovered diuretic agent that could improve the outcome of HF (39). Increases in the Na^+^/H^+^ exchanger may explain the phenomenon of the elevated Na^+^ level in HF (40). Ca^2+^ is required for cardiac diastolic function (41). In fact, Ca^2+^ signaling plays an essential role in regulating mitochondrial ATP production (42). Ca^2+^ is a second messenger in various cells and is regulated by ion channels, ion exchangers, pumps (ATPases), and Ca^2+^-binding proteins (43). The dysfunction of a sarcoplasmic reticulum Ca^2+^-release channel, ryanodine receptor, can cause calcium leakage and mitochondrial damage, which contribute to the progression of HF (44). Na^+^ is associated with Ca^2+^ uptake and Ca^2+^ related myofilament contraction through Na^+^/Ca^2+^ exchange (45).

### The Clinical Significance of Metabolic Remodeling: A Double-Edged Sword

Metabolic remodeling is a major pathophysiologic character of HF, but whether it is the cause or result of the HF, and whether it is maladaptive or adaptive is still controversial (46). Why have drugs both targeting inhibition and promotion of metabolic remodeling been used for the treatment of heart failure, and are both able to alleviate HF symptoms? FA or glucose, which is the superior energy substrate? We think that metabolic remodeling has a double effect: On one hand, metabolic remodeling is thought to be an adaptive compensatory mechanism. First, the shift toward glucose metabolism improves myocardial contractile efficiency by increasing the stoichiometric ratio of ATP production to oxygen consumption and reducing oxygen waste (47). Although glucose has a lower energy capacity, the shift is not due to a lack of substrate availability because the coronary circulation is able to provide an excess of substrates (47), and glycolysis produces ATP much faster than other ways, as epitomized by the Warburg effect (48). Second, similar metabolic remodeling can also be seen in the physiological remodeling of the heart. Many pathways, such as the activation of the AMPK and PI3K pathways, which have protective roles, are active in both physiological and pathological cardiac remodeling (20). On the other hand, metabolic remodeling is harmful when toxic substances such as accumulated excess intracellular FAs and ROS are increased, which may worsen HF and cause comorbidities. Recent evidence suggests that the accumulation of toxic intermediates, rather than alterations of substrate utilization or ATP deficit *per-second*, is responsible for cardiac dysfunction (18).

## Chronic Inflammation

### The Role of Inflammation in Heart Failure

HF is usually accompanied by highly elevated circulating pro-inflammatory cytokines, such as IL-1β, IL-6, IL-8, TNF-α, NF-κb, etc. However, the role of inflammation in HF has long been controversial. Because most traditional anti-inflammatory drugs failed in clinical HF therapies, inflammation was considered to not be a cause, but a complication of HF. The importance of inflammation in HF was not widely accepted until the success of canakinumab, an IL-1β inhibitor, which significantly improved the prognosis of HF. The effect of it and other anti-cytokine drugs indicates the role of inflammation in HF (49). Moreover, Soluble suppression of tumorigenesis-2(sST2) and galectin-3 are inflammatory biomarkers associated with fibrosis in HF, which have reportedly even better prognoses than NT-pro-BNP, an HF biomarker not directly associated with inflammation (50, 51). Having established the causal role of inflammation in HF, in the following, we give an overview of inflammation in cardiac remodeling and various comorbidities.

### The Immune Response Causes Systemic Inflammation

Both the innate and adaptive immune systems have a pro-inflammation role in HF. The immune response triggered inflammation mechanism is called immune inflammation. Innate immune cells, such as neutrophils, natural killer cells, and mast cells (52), have been revealed to participate in the progress of HF through immune inflammation. For instance, monocytes derived from HF patients have higher secreted cytokines (IL-1β, IL-6) and chemokines (CCL3, CCL4), and can stimulate T cell activation (53). Monocyte-derived macrophages have a pro-inflammation role in cardiovascular diseases (49). In addition, several pattern recognition receptors (PRRs), such as NOD-like receptors (NLRs) and Toll-like receptors (TLRs), are mainly expressed on tissue-resident immune cells, can turn on multiple signals to trigger innate immune inflammation. Finally, the activation of the innate immune system can cause the activation of the adaptive immune system by activation and infiltration of B cells and T cells (54).

### Inflammatory Cascade Promotes Cardiac Structural Remodeling

The major cardiac structural remodeling of HF including cardiac hypertrophy, fibrosis, and extracellular matrix (ECM) remodeling. Systemic inflammation can drive cardiac hypertrophy and fibrosis, and the inflammation is mainly triggered by PRRs (such as NLRP3 and TLR4) mediated innate immune response. The key inflammatory factors in this process are IL-1β, IL-6, and NF-κB. They can stimulate the release of many other inflammatory cytokines and transcription factors, which may promote cardiac hypertrophy and fibrosis (55, 56). The Nod1 receptor signaling pathway can contribute to cardiac hypertrophy (57). The mechanisms of the inflammatory cascade are yet not fully clear. However, it is known that IL-1β is activated by NLRP3 receptive inflammasomes. IL-1β and IL-6 stimulate immune cells (T cells, macrophages, and monocytes) to increase the release of IL-17, TNFα, and IFN-γ (58). These cytokines are signals which may activate immune cell trans differentiation into pro-inflammatory and pro-fibrotic subsets. For instance, Th1/Th2 polarization in T cells toward Th2 has a pro-fibrotic effect. While the CCR2+ monocytes, which express CC-chemokine ligand 2(CCL2) have pro-hypertrophy and pro-fibrotic effects (54). IL-33 is a member of the IL-1 family and ST2 is the receptor of IL-33. IL-33 has an anti-hypertrophic effect, whereas sST2 can competitively inhibit the IL-33/ST2 pathway and promote cardiac hypertrophy and fibrosis (59). Additionally, micro vascular inflammation can stimulate monocyte-derived macrophages to secrete transforming growth factorβ (TGF-β) which induces pro-fibrosis effects by stimulates the differentiation of fibroblasts into myofibroblasts. Myofibroblasts deposit collagen, and its increase may cause fibrosis (60). Moreover, inflammation may cause pyroptosis and apoptosis, which may also promote cardiac fibrosis (61).

The immune-inflammation mechanism may mediate cardiac ECM remodeling by increases ventricular stiffness (62, 63). Ventricular stiffness is a common pathological feature of HFpEF which promotes diastolic dysfunction (64). In systematic inflammation, IL-1β and other cytokines cause increased extracellular deposition of collagen and reduced elasticity of titin, resulting in ventricular stiffness (60).

## Reciprocal Promotion of Comorbidities and HF are Associated With Metabolism and Inflammation

Comorbidities and HF interact as both cause and effect, in which metabolism and inflammation are the possible common mechanisms underlying this cyclical relationship. In the following, some common comorbidities, including atrial fibrillation, diabetes, COPD, and obesity, are discussed from the standpoint of epidemiological evidence showing the reciprocal causation associated with underlying common metabolic and inflammatory mechanisms.

### Atrial Fibrillation

Atrial fibrillation (AF) frequently coexists with HFpEF and they share similar risk factors (65, 66). In a recent study, more than one-third of the AF patients had HF, and more than half of the HF patients had AF (67). Even subclinical AF was associated with about a 4-fold increase in HF risk (68). On the other hand, HF promotes AF via cardiac fibrosis, inflammation, and oxidative stress (69, 70). Cardiac resynchronization therapy with a defibrillator can reverse HF remodeling (71).

Metabolism and inflammation are the most consequential underlying mechanisms common to the two diseases. Cardiac energy alterations in HF cause subsequent oxidative stress and inflammatory cascades, and contribute to AF. Mitochondrial Ca^2+^ handling dysfunction is a shared mechanism in AF and HF, in which intracellular calcium leakage happens through oxidative stress-induced hyperphosphorylation of ryanodine receptor (43, 72). The PI3K/AKT may be a shared signaling pathway that regulates cardiac Ca^2+^ and Na^+^ ion channels (73).

### Diabetes Mellitus

The prevalence of type 2 diabetes mellitus (T2DM) in HF was about 20–50%, and T2DM may increase mortality due to HF (74). T2DM and HF coexist in about 30–40% of patients with T2DM (74–76). T1DM (77) is also associated with an increased risk of developing HF.

HF caused by coronary artery disease and hypertension secondary to T2DM is more common in HFrEF (74). Diabetic cardiomyopathy, which refers to HF occurring in the absence of related cardiovascular diseases, is generally believed to be mediated by abnormal mitochondrial calcium handling (78). HFpEF is also associated with insulin resistance-induced ventricular remodeling and mitochondrial dysfunction (79, 80). Chronic inflammation caused by excess insulin has also been found to be responsible for diabetic HFpEF (81). Moreover, the byproduct of glycolysis has recently been reported to link diabetes and HF by post-translational modifications (82, 83).

The molecular mechanisms underlying diabetic HF are associated with changes in myocardial substrate metabolism, inflammation, endoplasmic reticulum stress, aberrant insulin signaling, and autophagy (84). For one thing, hyperglycemia and insulin resistance cause excessive ROS production. Furthermore, oxidative stress causes chronic inflammation and mitochondrial metabolic disorders. Several molecular pathways are involved in these processes. ROS activates poly (ADP-ribose) polymerase (PARP) and inhibits the AMPK pathway and decreases mitochondrial biogenesis. These changes would cause disturbed circadian clock synchronization of glucose and FA metabolism. The insulin receptor may activate the PI3K/AKT pathway, which is a major mechanism responsible for insulin resistance induced cardiac dysfunction. Moreover, the activation of Na^+^/H^+^-exchange (NHE1/3) can promote HF (85). Finally, the NLRP3 inflammasome is activated in T2DM and triggers NLRP3/ IL-1β, IL-6, and IL-18 inflammatory pathways to contribute to cardiac fibrosis (86).

### Chronic Obstructive Pulmonary Disease

About 20% of unknown HF patients have COPD or asthma (87). Asthma increases HF risk by 80% (88). COPD is associated with increased risk (89) and worse prognosis of HF (90–92). The prevalence of systolic or diastolic HF in COPD patients ranges from 20% to 70% (93). Inhaled corticosteroid/long-acting β2-agonists (LABAs) in treating COPD were beneficial to cardiac function (94).

COPD may induce HF through chronic systemic inflammation and pulmonary vascular remodeling (95). In turn, HF aggravates excess ventilation in COPD, and causes dyspnea, and exercise intolerance (96).

### Obesity

The prevalence of obesity in HF was about 40% (97, 98). Obesity increases the risk of HF (99). However, there is a U-shaped relationship between BMI and survival of HF - the so-called “obesity paradox.” That is, high BMI is associated with better survival in patients with HF. However, the mortality risk from HF increased for patients with extremely high BMIs of 45 or greater (98). Furthermore, high waist-to-hip ratios have been associated with increased mortality, suggesting the harmfulness of obesity in HF (100). Abdominal obesity is associated with significantly higher mortality in HFpEF, which may be a better predictor than BMI (101). Abdominal obesity is strongly associated with the circulating level of aldosterone, the main role of which is to regulate salt-water retention. Mineralocorticoid receptor antagonists have recently been discovered as targets for obesity-associated HF (102).

Metabolism and inflammation are involved in the progress of HF in patients with obesity (103, 104). Increased leptin, which is reported as the product of the obesity gene, contributes to cardiac remodeling through Leptin-Aldosterone-Neprilysin Axis (105, 106). Insulin resistance secondary to obesity can cause altered cardiac energy metabolism and HF (107, 108). Obesity can cause immune-inflammation by activating macrophages, and activate IL-1β and NF-κB pathway (104).

Obesity can suppress BNP levels in HF (109) and causing lower plasma NT-pro-BNP levels (5). Therefore, BNP may not reflect the HF severity accurately in obese patients (110, 111). BNP enacts cardiac protection via multiple actions, such as suppressing RAAS activation and regulating sodium metabolism. An insufficient BNP level may promote HF progression (112).

### Cancers

Cancers and HF are often coexisting in patients with cancers, they share several common pathophysiological mechanisms and causes, such as angiogenesis, clonal haematopoiesis, and sarcopenia (113–115). Aging may cause somatic mutations of genes (typically DNMT3A and TET2) in hematopoietic stem cells, which promote peripheral blood leukocytes release proinflammatory factors such as IL-1β and IL-6. This phenomenon is called clonal hematopoiesis of indeterminate potential (CHIP), which is a risk factor of cardiovascular diseases and cancer (116). Sarcopenia is a common complication in advanced stage cancer, which may promote HF through muscle wasting and thinning of the ventricular wall, (115).

Cardio toxicity is a major risk factor for HF. It reportedly accounts for 45% of all drug withdrawals (117). Mitochondrial dysfunction is the major pathophysiologic mechanism of drug-induced cardio toxicity (117, 118). In most times a drug with cardio toxicity would not be used in clinical. However, anti-tumor drugs with cardio toxicity are common when weighing the pros and cons because of the therapeutic effect (119). For instance, aromatase inhibitors have become the preferred treatment for estrogen receptor-positive breast cancer, which targets the cytochrome P450 enzyme, but it is associated with a significantly increased risk of HF (120). Anthracyclines such as doxorubicin (121) and epirubicin (122) are commonly used for breast cancers, lymphoma (123), and a variety of other cancers, but their usage is limited by cardio toxicity. Trastuzumab, another breast cancer drug, is also associated with increased HF risk (124). The proposed biological mechanisms underlying anthracycline cardio toxicity are mitochondrial dysfunction, mitochondrial iron overload, oxidative stress, inflammation, and impaired autophagy (125).

## Link Between Heart Failure and Comorbidities

Common risk factors such as aging, hyperglycemia, and lifestyle are the cause of HF and comorbidities. The underlying mechanisms of these factors are associated with common metabolic or inflammatory pathways. In this review, the major pathways were identified through gene enrichment analysis. Further, the common therapy drug targets have also be summarized by analyzing the disease-gene network. This review will be helpful for selecting the therapeutic strategy.

### Major Shared Risk Factors of HF and Comorbidities Are Associated With Metabolism and Inflammation

Epidemiologic evidence has found many risk factors for cardiovascular diseases, including chronic conditions or diseases (aging, hyperlipidemia, hypertension, hypoxaemia, and metabolic syndrome), and lifestyles (dietary and sleeping patterns, smoking, and drinking (126–128). Unhealthy lifestyles may contribute to HF by dysregulated innate immunity and chronic inflammation (129). These factors are also risk factors for many comorbidities (130) and share similar mechanisms, which are associated with metabolism and inflammation.

Aging is one of the major risk factors for developing multi morbidity and HFpEF, and both multi morbidity and HFpEF are unmet needs in the therapy of HF (131–133). The main underlying mechanisms of cardiovascular aging are associated with mitochondrial metabolism (134, 135), chronic inflammation (136), autophagy (137), and oxidative stress (138).

Physical inactivity (sedentary behavior), is a risk factor of multi morbidity (139), it causes chronic subclinical myocardial injury detectable with high-sensitivity cardiac troponin and increases HF risk (140). Meta-analysis showed exercise is beneficial for people with multi morbidity (141). It can regulate mitochondrial remodeling (142), and also causes physiologic remodeling which increases cardiorespiratory fitness (143). It is improved cardiorespiratory fitness that is the physiopathological link between obesity, exercise, and HF (93, 94), primarily by increases the cardiac compensatory capacity (17). Furthermore, exercise has direct anti-inflammatory effects by inhibition of TNF-α and IL-1β, and may attenuate insulin resistance (144).

Metabolic syndrome, mainly charactered by hyperlipidemia and hypertension, shared similar mechanisms to that of diabetes and obesity, such as insulin resistance and macrophage induced inflammation, which have already been discussed (104). Taken together, metabolism and chronic inflammation are the major mechanisms underlying the major shared risk factors between HF and comorbidities.

### Common Molecular Pathways Analysis

**Figure 3:**
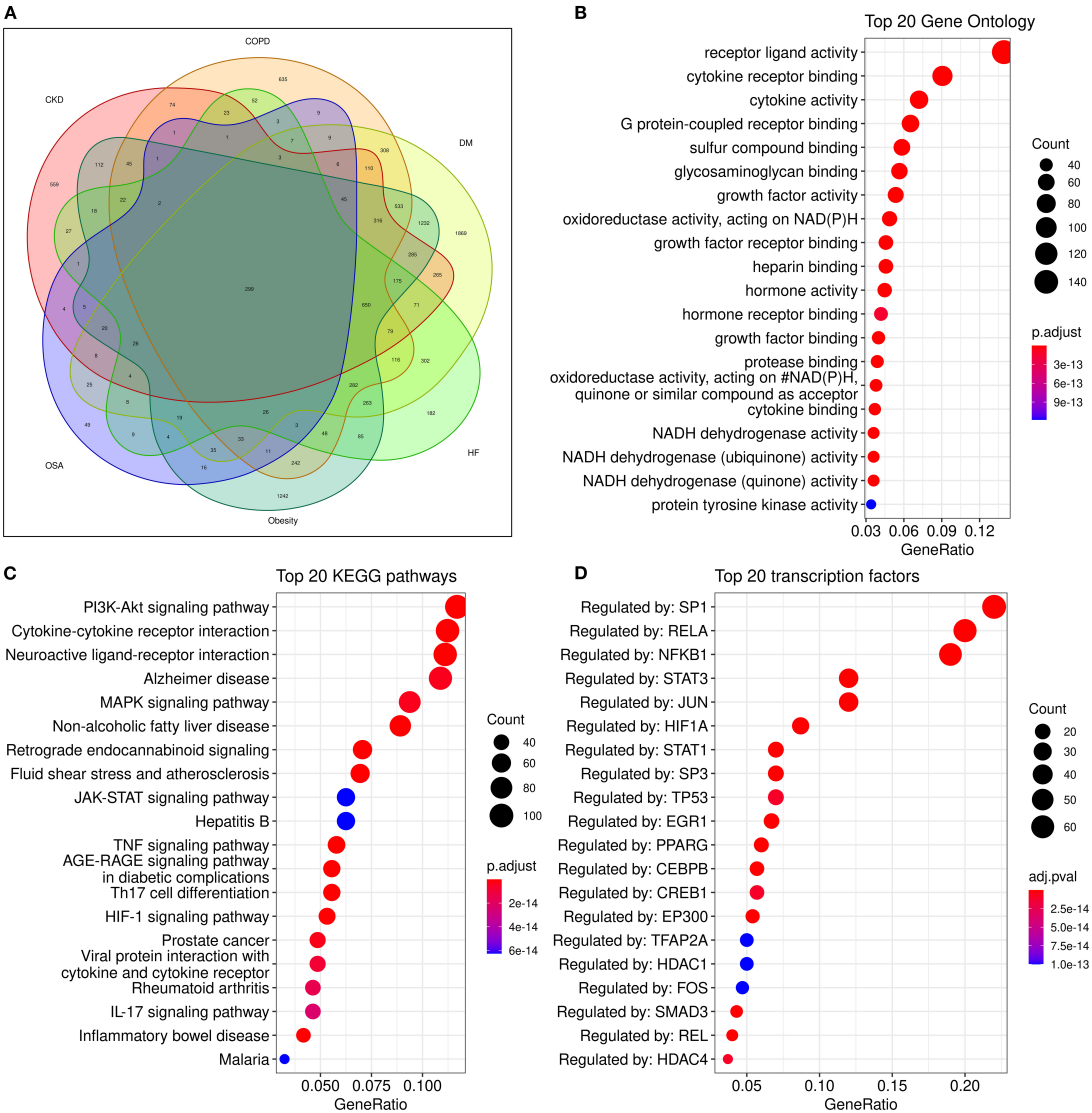
Analysis of common genes and pathways in comorbidities and HF. **(A)**: Venn diagram of common genes between comorbidities and HF; **(B)**: Dot plot of top 20 enriched Gene Ontology biological processes enriched for common genes between HF and comorbidities; **(C)**: Dot plot of top 20 enriched KEGG (Kyoto Encyclopedia of Genes and Genomes) pathways for common genes between HF and comorbidities; **(D)**:Dot plot of top 20 enriched transcription factors from TRRUST(Transcriptional Regulatory Relationships Unraveled by Sentence-based Text mining) database.

Although many metabolism and inflammation mechanisms have been reviewed previously, which pathways are most important remains unclear. To conduct an unbiased analysis of the key shared biological pathways in HF and comorbidities, we performed enrichment analysis on target genes of HF and some comorbidities of high prevalence in the database. The Target Validation platform (https://www.targetvalidation.org/) contains disease target genes from Genome-Wide Association Studies (GWAS), drug targets from the EMBL-EBI ChEMBL database, EMBL-EBI RNA expression data, and text mining of literature. First, we retrieved all the targets of HF and several comorbidities (diabetes mellitus, obesity, COPD, chronic kidney disease, and OSA) in the Target Validation platform (accessed on March 22, 2021) and intersected the disease targets as shown in the Venn diagram (Figure 3A). Five comorbidities (diabetes mellitus, obesity, COPD, CKD, and obstructive sleep apnea) were selected for analysis because these represent the most highly prevalent comorbidities (The major enriched pathways did not change but the Venn diagram and the latter network plot would be more complex and less understandable when adding other common comorbidities such as atrial fibrillation and depression into the analysis). There were 299 common targets associated with all the four diseases, and 1,051 common targets were shared by HF and at least four of the comorbidities. Gene Ontology and KEGG enrichment analysis was performed on 1,051 semi-common targets with the R (version 3.6.0) package cluster Profiler (version 3.14.3). The activation of metabolic and inflammatory pathways may require the expression level change or activation of a group of enzymes, cytokines, or proteins regulated by common transcription factors. To identify key transcription factors, transcription factors enrichment analysis was performed using Meta scape website tools (http://metascape.org) (145) with TRRUST (Transcriptional Regulatory Relationships Unraveled by Sentence-based Text mining) database (146) and the figure was plotted with ggplot2 (version 3.3.3).

Some known factors which play a crucial role in HF, such as the NADPH oxidase (147), and sulfur compound binding (148), and growth factor activity (149) were enriched in the Geno ontology enrich analysis (Figure 3B).

The enriched pathways are mainly associated with metabolism and inflammation. Some significantly enriched pathways not shown in the figure are also analyzed. According to their role in HF, most of the significantly enriched pathways can be classified into one or more of the following categories: (1) Energy metabolic associated pathways. The PI3K/Akt pathway regulates both metabolic and structural remodeling. The PI3K/AKT pathway is associated with AF (150), COPD (151), HF (152), and multi morbidity (131). The PI3K/AKT pathway regulates cardiac metabolism both in pathological remodeling in HF (143), and it also regulates heart growth (149). (2) Structure remodeling associated pathways. The MAPK pathway is the key pathway activated in response to ischemia and has a critical role in cardiac hypertrophy. Moreover, the MAPK pathway may be involved in the interplay of mitochondrial energy metabolism and systemic inflammation (57). The Hypoxia-Inducible Factor 1 (HIF-1) pathway can regulate glucose metabolism and is adaptively activated in response to hypoxia conditions and can promote cardiac hypertrophy. HIF-1 can activate the glycation end products (AGE) advanced glycation end products (RAGE) signaling. The AGE-RAGE signaling pathway is associated with some comorbidities of HF such as OSA and diabetes (153, 154). Insulin-like growth factor (IGF) signaling is activated in HF and promotes cardiac hypertrophy (155, 156); (3) Cardiac systolic and diastolic functions associated pathways. Such as the CaMKII pathway (157), The G protein-coupled receptor (GPCR) signaling pathway is a known drug target of HF, these drugs include β-adrenergic receptor and angiotensin II receptor antagonists (158). (4) Inflammatory pathways, majorly include the Cytokine-cytokine receptor interaction, TNF signaling, IL-17 signaling, and Toll-like receptor pathways (Figure 3C). Additionally, the clonal hematopoiesis pathway is a risk factor of HF enriched in the analysis and may be related to immune inflammation (53, 159). (5) Other structural remodeling in addition to hypertrophy, such as fibrosis and amyloidosis. The activation of inflammatory pathways can activate the TGF-β pathway and promote fibrosis. Alzheimer's disease is also enriched. Alzheimer's disease is major characterized by amyloidosis, and senile amyloidosis may be an overlooked causal mechanism of HFpEF (60, 66). The PI3K/AKT/GSK3β pathway is proposed as the link between diabetes and Alzheimer's disease (160).

The PI3K/AKT pathway is most significant in the pathway enrich analysis and is a key pathway in cardiac remodeling. Cardiac remodeling is a key biological process that contributes to the progression of HF. Some drugs, such as calcium antagonists and renin inhibitors, may alleviate hypertension and improve contraction function of HF, they did not improve remodeling, and therefore did not improve the prognosis of HF (161). Some downstream signaling pathways, such as PI3K/AKT/eNOS have a cardio protective role, and the activation of this pathway may be the mechanism of some cardiovascular drugs such as statins (162). PI3K/AKT pathway activation is a shared mechanism in physiological and pathological cardiac hypertrophy, and physiological hypertrophy may enhance cardiac systolic and diastolic function (143). However, in pathological conditions, such as HF, long-term sustained activation of PI3K/AKT pathway in HF promotes excessive cardiac growth, mitochondrial dysfunction, ROS production, and impaired Ca2^+^ handling (163). Activation of PI3K/AKT pathway is a common mechanism in many chronic diseases, such as cardiovascular disease, metabolic diseases, COPD, and cancers (131). It has been reported improved HF syndrome with no substantial side effects when using PI3K/AKT inhibitors as a treatment of PIK3CA-related overgrowth syndrome (164). Therefore, PI3K/AKT inhibitors may be a promising treatment for HF and comorbidities. However, because the activation of PI3K/AKT pathway is essential for many cellular processes such as cell growth, proliferation, and migration, targeting PI3K/AKT pathway may have side effects, finding a more specific target of HF and comorbidities related to PI3K/AKT pathway may be a better treatment choice.

### Common Mechanistic Pathways in Heart Failure and Comorbidities

**Figure 4:**
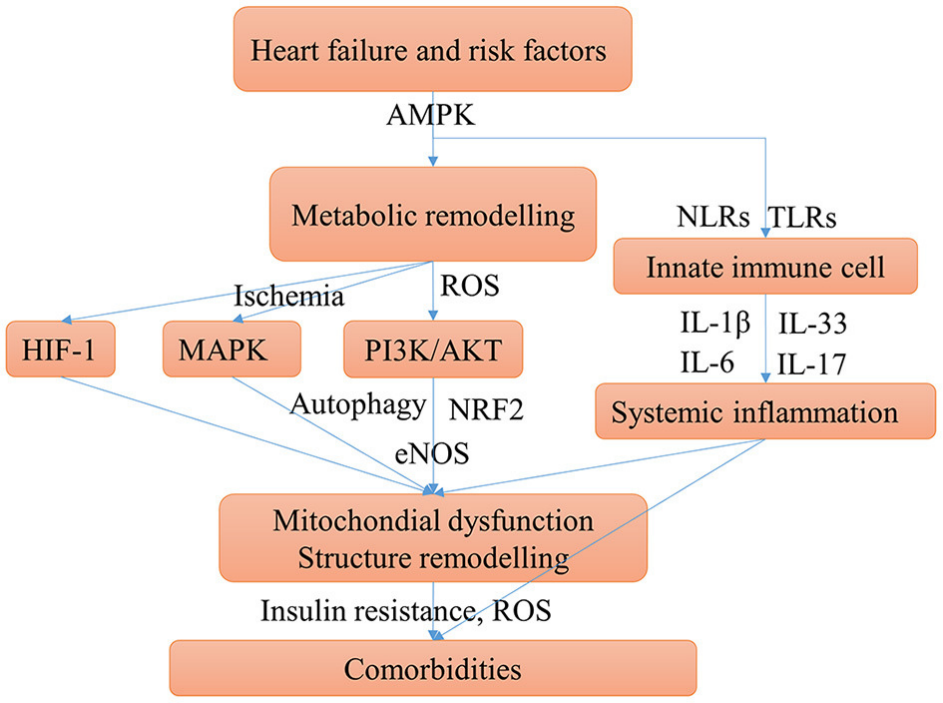
Main possible shared mechanisms underlying HF and comorbidities. HF, comorbidities, and risk factors may have some shared chronic conditions such as insulin resistance, hypoxia, and chronic inflammation, these conditions aggravate HF and cause comorbidities. The mechanism of comorbidities contribute to HF can be clarified similarly. Cardiac energy metabolic remodeling may take place in these conditions mediated by activating the AMPK signaling. Metabolic remodeling can activate PI3K/AKT pathway, which promotes myocardial over-growth and cardiac hypertrophy and can cause mitochondrial injury. The mTOR signaling may be activated by AMPK and PI3K/AKT pathways can cause disturbed autophagy which aggravates the mitochondrial injury. Ischemia and hypoxia conditions can activate MAPK and HIF-1 pathways, which contribute to cardiac structure remodeling. Innate immune cells, mainly monocytes, macrophages, and neutrophils, can trigger the immune response and systemic inflammation by secreting IL-1β and IL-6. Moreover, the pro-inflammatory cytokines stimulate T cells to polarize to Th17 cells and release IL-17. Systemic inflammation can cause diastolic dysfunction and cardiac hypertrophy.

Together with a review of the literature into account, the main shared mechanisms of HF-induced comorbidities can be summarized (Figure 4) and elucidated. The mechanism of how comorbidities promote HF may be clarified similarly by the shared mechanisms.

The metabolic mechanisms of HF promote comorbidities are associated with mitochondria injury, oxidative stress, insulin resistance, and hypoxia. HF and risk factors induce altered cardiac energy metabolism. Cardiac energy metabolic remodeling causes oxidative stress through NAD (P) H oxidase-derived ROS (165). Oxidative stress can trigger mitochondrial injury and inflammation. As such, antioxidants have been a therapeutic strategy for cardiovascular diseases (166). Oxidative stress, mitochondrial dysfunction, and chronic inflammation were the major mechanisms of multi morbidity in the elderly (131). There is a consensus that mitochondrial impairment is key to cardiac dysfunction in HF (167). Mitochondria injury can cause cardiac remodeling, such as hypertrophy and fibrosis (168). In addition, mitochondrial biogenesis dysfunction play important roles in multi morbidity such as diabetes (169), obesity (170), lung diseases (171, 172), depression (173), sarcopenia (142), iron deficiency (148, 174), fatty liver disease (175), obstructive sleep apnea (176), and diabetic kidney disease (177). Mitochondria injury is commonly induced by oxidative stress or inflammation mediated by the PI3K/AKT/eNOS, PI3K/AKT/mTOR, AMPK/mTOR pathway (178), or the MAPK signaling pathway (179). Mitochondria autophagy, also called mitophagy, is a cellular process in which impaired mitochondria are destroyed to protect eukaryotic cells from mitochondrial injury. Autophagy has a protective role for HF and comorbidities, and may be injured by the activation of mTOR pathway (180). Insulin resistance plays an important role in the pathological processes of HF, and is also strongly associated with diabetes (181), as well as obesity in which is associated with the phosphorylation of PPARγ (182). Insulin resistance was associated with the worse outcomes in patients with HF and diabetes (183). Hypoxia is a common chronic condition in many comorbidities such as COPD and anemia, and the related HIF-1 pathway may have an important role in the progression of obesity and hypertension (104).

Chronic systemic inflammation associated with HF is mainly triggered by innate immune cells (monocyte, macrophage, and neutrophils). The major pro-inflammatory cytokines including IL-1β, IL-6, IL-8, IL-17, IL-18, and TNA-α (49, 184, 185). Apart from their role in HF, IL-1β and IL-6 are key pro-inflammatory factors in many diseases, like COPD (186), diabetes (187), kidney disease (188), sarcopenia, obesity, and HF (189), and the cytokine storm in COVID-19 (190). A recent study on HFpEF supported that systemic inflammation may be the association between comorbidity and HF (191). The IL-1β and IL-18 signaling pathways may be novel drug targets for HFpEF, which are important in the mitochondria-inflammation circuit (192).

The alteration of pathways is often regulated by transcription factors as switches. Many common transcription factors have been found including SP-1, RELA, NF-κB, STAT3, HIF-1α, PPARγ, c-FOS, and c-JUN (Figure 3D) and together with a review of the literature, the transcription factors network in HF and comorbidities are briefly summarized as follows: (1) Regulation of inflammation. NF-κB is the key transcription factor in inflammation. Both RELA and NFKB1 are genes of NF-κB subunit. NF-κB regulates inflammation initiated Ca^2+^/Calmodulin-dependent cardiac remodeling (193). STAT3 is a predicted target regulated by NF-κB in Figure 3D. The activation of NF-κB and STAT3 is required for the expression of multiple inflammatory cytokines including IL-1β (194), TNA-α (195) and IL-6. The c-FOS and c-JUN are family of AP-1, which regulate the MAPK pathway, and can be inhibited by SIRT3 (196). EGR1 and c-FOS are also associated with the release of IL-1β (197). SP-1 can regulate immune responses, but it is a non-specific transcription factor involved in many other cellular processes and indicates transcriptional activation; (2) Regulation of metabolism. The activation of PPARγ is essential for the FAO process (18). The sirtuin family members SIRT1, SIRT2, and SIRT3 are important transcription factors in cardiac energy metabolism and have similar roles. SIRT3 regulates ATP production (198). SIRT2 and PPARα regulate glycose metabolism by the AMPK pathway (199), SIRT1 and NRF2 regulate energy metabolism and mitochondrial biogenesis (200).

### Common Therapeutic Drug Targets

**Figure 5:**
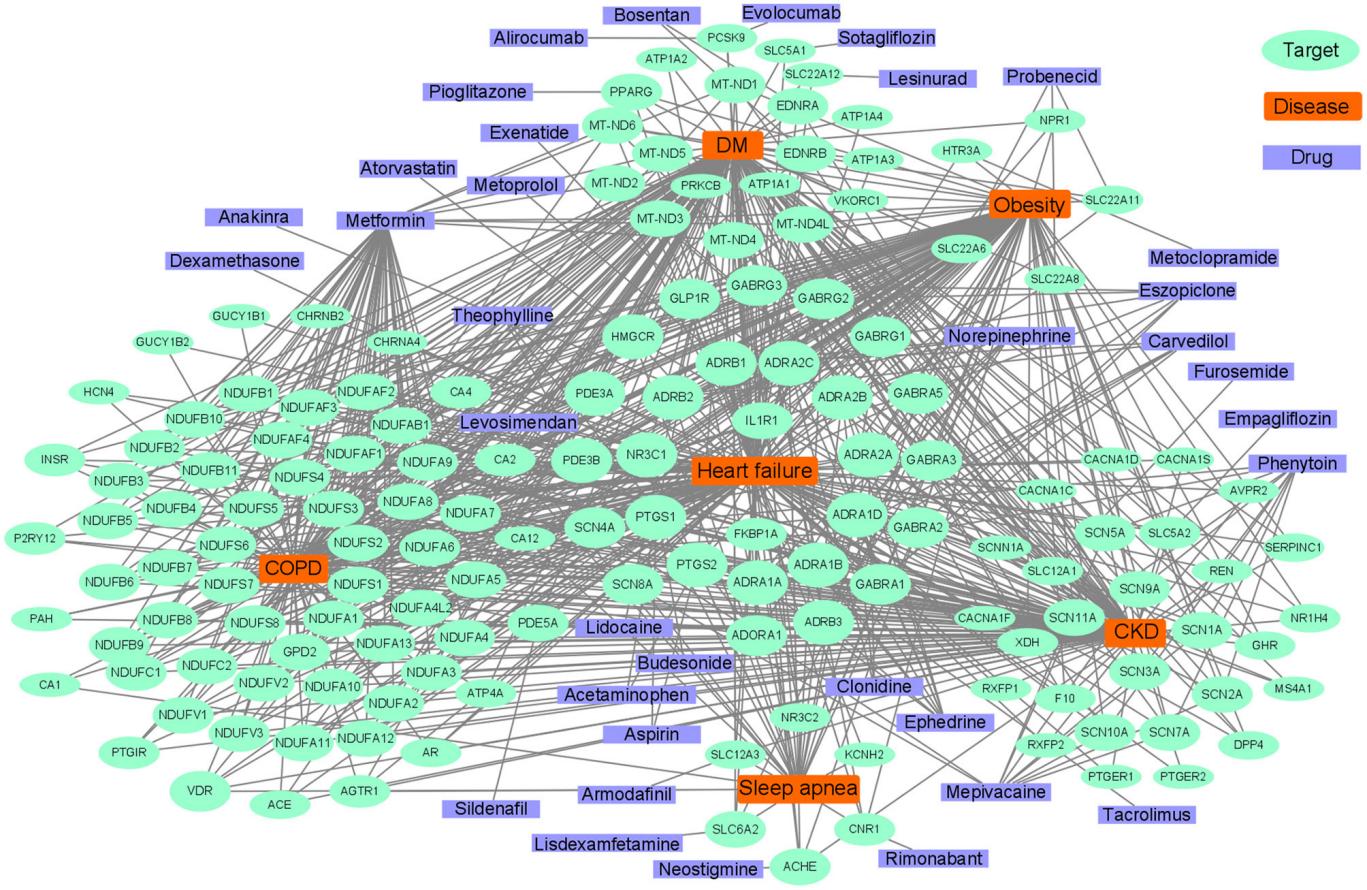
Abridged common drug target network of heart failure and comorbidities.

Common pathways indicate common targets, which are the basis for drug repurposing. Network analysis is often used in the repurposing of drugs (201). The known drug targets of HF, diabetes mellitus, COPD, CKD, sleep apnea, and obesity were retrieved from the Target Validation Platform (targetvalidation.org). We constructed a disease-target network in Cytoscape 3.8.0 (202). Some representative drugs were randomly chosen and listed in Figure 5 to provide an example. The drugs range from old drugs like metformin to relatively new ones in HF treatment, like SGLT2 inhibitors. However, network analysis has some limitations and should be interpreted combined with literature review. For one thing, it is based on the database, and some drugs in the database had been investigated in HF clinical trials but have no effect. Some drugs such as calcium channel blockers could not treat HF. For another, a drug associated with multiple targets might be non-specific and does not necessarily have better effects. For instance, doxorubicin inhibits both Top2a and Top2b, inhibiting Top2a have an anti-cancer effect while inhibiting Top2b have a cardiac side effect (125). Anti-inflammatory therapy with Canakinumab (203) in clinical trials which target IL-1β can reduce the mortality of HF patients. IL-1β is an important inflammatory cytokine associated with many comorbidities. Canakinumab can improve the prognosis of cardiovascular outcomes in patients with CKD (204). However, Canakinumab could not reduce the incidence of new-onset diabetes (205), which suggests the role of inflammation in diabetes might be less important. Anakinra, a recombinant IL-1 receptor antagonist, is another drug targets IL-1β, it is under phase III clinical trial in HF and has a therapeutic effect (206). In summary, IL-1β inhibitors/antagonists are promising drugs for HF and comorbidities.

Diabetes drugs are a good example of drug repurposing applied in HF. Some therapy of diabetes may increase the risk of HF such as insulin (183), whereas some drugs such as metformin, sulphonylureas, and gliptins either alone or in combination, could significantly reduce the risk of HF (207). The SGLT2 inhibitors are originally designed for diabetes, which targets the SLC5A2 gene, and have shown benefit for HF, regardless of whether comorbid with diabetes or not (208, 209). In a clinical trial, there were unexpected excellent risk reductions in hospitalization for HF and all-cause mortality with the use of the SGLT2 inhibitor, empagliflozin (210). The benefit of empagliflozin could not be explained by the effects of classical inhibitors, such as natriuresis or neurohormonal mechanisms. It has been speculated that the shift in cardiac energy substrate may play a major role in the cardiorenal benefits of empagliflozin; that is, a shift from using glucose and fat to ketone bodies (211). Linagliptin, a DPP-4 inhibitor designed to treat diabetes, can also be used to treat HF (212, 213). Metformin affects many targets that are associated with oxidative phosphorylation in mitochondria (214), such as MT-ND5 and NDUFB7, and has been reported to have therapeutic effects on HF and comorbidities. Metformin is an indirect AMPK pathway activator, and also increases glucose transport and catabolism by increasing the residence time of GLUT4. AMPK agonists are promising HF therapy drugs, which consist of direct activators, such as A-769662 (a preclinical drug), or indirect activators, such as 5′-aminoimidazole-4-carboxyamide-ribonucleoside (22, 215).

Although many anti-tumor drugs have cardio toxicity, network analysis of shared pathways and targets enables us to find drugs beneficial for both diseases. For example, PI3K/Akt/mTOR pathway is a shared pathway in cancers and HF, drugs targeting the mTOR pathway, such as rapamycin, are novel potential drugs for HF which can reduce cardiac remodeling and HF (119).

There are some genes of the phosphodiesterase family, such as PDE5A, PDE3A, and PDE3B. PDE5 inhibitors (such as Sildenafil) regulates the nitric oxide synthases and hydrogen sulfide (H2S) generation, and may attenuate ROS induced mitochondrial dysfunction through the AMPK pathway (216). However, side effects largely limit its clinical application, probably because PDE5 is involved in a variety of biological processes not specific to HF.

Beyond known drug targets, some targets may have similar functions as they belong to the same protein family. Similar to SLC5A2, SLC25A51 is a member of the solute carrier family, and has been recently found to be a mitochondrial NAD+ transporter (217) and may perhaps serve as a new drug target.

### Links Between Heart Failure Phenotypes and Comorbidity

Multi morbidity and HFpEF are both unmet needs in HF therapy. Comorbidities exist in both HFpEF and HFrEF, but the prevalence of most comorbidities is higher in the HFpEF than reduced ejection fraction (HFrEF) (6, 218), indicating a strong association between HFpEF and comorbidities (6). The prevalence of preserved ejection fraction HF (HFpEF) is rising, and mortality remains high because of the absence of effective therapies (60, 219), which gives rise to the urgent need for drug discovery targeting HFpEF. Although HFpEF has a better ejection fraction than HFrEF, the mortalities are similar, and the higher frequency of morbidities in HFpEF than HFrEF may explain the phenomena (220). The risk factors and incidence of comorbidities are different, therefore the pathways, therapeutic targets, and drugs between the subclasses of HF were different. COPD and OSA are associated with increased HFpEF disease risk and adversely impact cardiovascular disease outcomes, in which chronic inflammation and oxidative stress are responsible for the association. Therefore, drugs like statin and/or antioxidants may be beneficial (221, 222). Compared with HFrEF, there are more hypertension and fewer coronary diseases in HFpEF (218, 223). Atrial fibrillation is associated with significantly increased mortality (224), and AF is more frequently in HFpEF than HFrEF (218). Because multi morbidity is more frequent in HFpEF, targeting the common pathways between comorbidities may be a potential novel therapy for HFpEF.

Expression levels of biomarkers is also different between systolic and diastolic HF. The BNP level is lower in HFpEF (225) and NT-proBNP/BNP-guided therapy was reportedly only beneficial in HFrEF because comorbidities may influence BNP level and provide misleading information (226).

## Conclusion and Future Perspectives

In this review, we concluded the pathology and molecular mechanisms of comorbidities of HF. Metabolism remodeling and chronic inflammation are responsible for the major underlying pathophysiologic links between HF and comorbidities. Mitochondrial metabolism is expected to play a central role, but no drugs specifically conceived to modulate mitochondrial functions are currently available (227). The therapy for comorbidities of HF is increasingly becoming challenging. The common metabolic and inflammatory mechanisms may provide promising possible therapeutic targets for both HF and comorbidities, which may be useful for both old drug repurposing and the discovery of new drugs.

## Author Contributions

ZL performed an extensive literature review, drafted the manuscript, and prepared figures. JW and HZ proposed the subject of the review, critically revised, and edited the manuscript. All authors contributed to the article and approved the submitted version.

## Conflict of Interest

The authors declare that the research was conducted in the absence of any commercial or financial relationships that could be construed as a potential conflict of interest.
